# Ankle muscle strength and activation are associated with walking patterns in preschool and school-age children

**DOI:** 10.1371/journal.pone.0316826

**Published:** 2025-01-14

**Authors:** Sudarat Apibantaweesakul, Shiho Omura, Weihuang Qi, Natsuki Sado, Hiroto Shiotani, Fumiko Tanaka, Plaiwan Suttanon, Yasuo Kawakami

**Affiliations:** 1 Graduate School of Sport Sciences, Waseda University, Saitama, Japan; 2 Faculty of Allied Health Sciences, Thammasat University, Pathum Thani, Thailand; 3 Waseda Institute for Sport Sciences, Waseda University, Saitama, Japan; 4 Toho-Leo Co., Tokyo, Japan; 5 Institute of Health and Sport Sciences, University of Tsukuba, Ibaraki, Japan; 6 Faculty of Sport Sciences, Waseda University, Saitama, Japan; 7 Human Performance Laboratory, Comprehensive Research Organization, Waseda University, Tokyo, Japan; 8 Thammasat University Research Unit in Health, Physical Performance, Movement, and Quality of Life for Longevity Society, Thammasat University, Pathum Thani, Thailand; Ningbo University, CHINA

## Abstract

Walking patterns can differ between children and adults, both kinematically and kinetically. However, the detailed nature of the ankle pattern has not been clarified. We investigated musculature, biomechanics, and muscle activation strategies and their relevance to walking performance in preschool (PS) and school children (SC), with adults (AD) as reference. Twenty-six PS (3–5 yr), 20 SC (6–8 yr), and 17 AD (18–30 yr) participated. Tibialis anterior (TA) and medial gastrocnemius (MG) thicknesses, fascicle lengths, and maximal voluntary dorsi- and plantar flexion isometric torques were measured. Hip, knee, and ankle kinematics, ground reaction forces (GRFs), and TA and MG electromyographic activities were recorded during shod walking at each participant’s preferred speed. Walking speed, step length, and cadence were correlated with age in PS. These walking performance measures were also correlated with muscle thickness and fascicle length, showing higher speed in individuals with thicker muscles and longer TA and MG fascicles (conversely, higher cadence with thinner muscles and shorter fascicles). AD demonstrated the largest values for muscle thickness (*p*<0.001), fascicle length (*p*<0.001), strength (*p*<0.001), and walking performance (speed *p* = 0.004; step length p<0.001; cadence *p*<0.001), followed by SC then PS. Both PS and SC exhibited higher TA activities than AD during the stance phase, resulting in a higher co-activation index. The GRFs relative to body weight were lower in both horizontal and vertical components in PS compared to SC and AD, while the relative lateral force during stance was greatest in PS followed by SC and then AD. Differences in preferred walking speed and step length were associated with age and muscle size. Children, particularly preschool-aged, employed a co-activation strategy of dorsi- and plantar flexors for stabilization, which resulted in sideways steps even at a preferred walking speed.

## Introduction

Human bipedal walking is known to be energetically efficient, achieved through inverted pendulum-like actions of the lower extremities, which function as springs [1]. During a single walking cycle, each step comprises the stance and swing phases, enabling the body’s forward propulsion through three stages of lower limb movement: weight transition, single-limb balance, and limb advancement. The stance phase accounts for approximately 60% of the gait cycle and involves functions such as single-leg balance, support of total body weight on the contact limb, forward propulsion, and directional control of the swing leg [2, 3]. These tasks are achieved to a greater part through movements of the hip, knee, and ankle joints [2, 4–6].

Walking patterns are known to differ between adults and children, with notable distinctions in ankle moment across age groups; older children, for example, demonstrate a higher maximum ankle moment than younger children [7]. In adults, the ankle dynamically absorbs shock and generates propulsive forces during the stance phase [2, 4, 6]. A previous study addressed the primary roles of muscles around the ankle (dorsi- and plantar flexors) in weight acceptance and propulsion during stance [5]. There is evidence showing that maturation of walking is related to ankle biomechanics [6, 8–10], activation patterns of dorsi- and plantar flexors [5], and musculoskeletal growth [11, 12]. Regarding muscle morphology, muscle volume represents the three-dimensional muscle size including muscle length and cross-sectional area, or thickness in sagittal and frontal planes, as assessed through medical imaging [13]. As bones lengthen during growth, muscles and tendons elongate as well [14]. For clinical assessment, the muscle volume might be limited, therefore, muscle thickness is often measured as a proxy for muscle growth, showing age-related increases in the tibialis anterior (TA) and gastrocnemius muscle [11, 12]. Previous studies have shown a positive relationship between muscle size and strength in both children and adults [15–17], and the muscle architectural characteristics (including fascicle length and pennation angle) have been linked to locomotion performance [18, 19]. Additionally, body dimensions have been identified as factors influencing walking performance [20].

Early childhood is often divided into two groups, preschool (3–5 years; PS) and school children (6–8 years; SC) that have different growth rates and walking performance [21]. Recently, we showed that muscle morphology and strength of anterior and posterior lower leg muscles in absolute terms substantially differed between PS and SC [6, 20]. However, previous studies did not address specific aspects including anatomical and motor control factors in each age group that may influence walking performance. Furthermore, the biomechanics of walking—particularly ankle kinematics and kinetics in children—remains a topic of debate, with studies showing significant variability in ankle joint maturation, with kinematic changes observed from ages 5 to 13 and kinetic changes from ages 3 to 8 [6].

Based on these morphological growth and strength developments, we hypothesize that preschool children exhibit significant differences in muscle thickness, fascicle length, and gait patterns compared to school-aged children and adults, potentially reflecting developmental changes in motor control strategies and walking performance. To test these hypotheses, we examined lower leg muscle characteristics, biomechanical parameters of shod walking, and muscle activation profiles in PS and SC, with adults (AD) as a reference group.

## Methods

### Study design

A cross-sectional study was conducted. The recruitment period was from October 20, 2018, to July 21, 2019. The study included ultrasonography and strength measurements, after which each participant was asked to walk along an 8-meter flat walkway in the laboratory at their self-selected, comfortable walking speed. Participants wore shoes with appropriate size, manufactured by ASICS Corporation (ASICS Idaho Baby 3, foot length 14.0–15.0 cm; ASICS Idaho Mini 3, foot length 16.0–22.0 cm; and ASICS FuzeX Rush Adapt, foot length ≥ 23.0 cm). Kinematics, ground reaction forces (GRFs), and muscle activity were recorded during walking. Participants were allowed to practice with their familiarized walking 2 times. Then, each walking session was performed with 30 seconds of rest between trials. A minimum of three walking trials with clean foot strikes on the force plate were obtained with encouragement to keep the movement as stable as possible.

### Participants

Twenty-six preschool children (PS; 20 boys and 6 girls), and 20 school children (SC; 11 boys and 9 girls) voluntarily participated in this observational cross-sectional study. Seventeen adults (AD: 20–35 years; 12 men and 5 women) were included as a reference group. Participants were assigned to groups based on age criteria (PS: 3–5 years SC: 6–8 years AD: 20–35 years) and inclusion/exclusion criteria. Participant characteristics are presented in Table 1. The study was approved by the Human Research Ethics Committee of Waseda University (reference number: 2017–233). Written consent/assent, in accordance with the Declaration of Helsinki, was signed by all participants or their parents/guardians prior to participation. Participants were excluded if they had any chronic disorders, lower extremity injuries, or were on continuous medication.

**Table 1:** Anthropometric, and lower leg morphological and strength parameters.

	Preschool children		School children		Adults		*p*-value	Sig. (Post hoc)	Cohen’s d	95%C.I.
	Median	IQR	Median	IQR	Median	IQR				
Age (years)	4.7	1.5	7.1	1.9	24.5	5.0	<0.001	abc	-10.21	-12.07, -8.34
Body height (cm)	104.5	9.7	122.4	11.4	171.1	8.4	<0.001	abc	-8.23	-9.77, -6.69
Body mass (kg)	16.5	3.2	23.4	7.0	59.9	9.8	<0.001	abc	-8.26	-9.80, -6.71
BMI (kg/m^2^)	15.3	1.5	15.4	2.5	20.9	3.3	<0.001	bc	-3.28	-4.07, -2.48
TA muscle thickness (mm)	17.0	2.1	18.5	3.6	29.2	3.4	<0.001	abc	-6.46	-7.72, -5.21
MG muscle thickness (mm)	12.4	2.1	14.8	2.7	20.5	4.3	<0.001	abc	-3.60	-4.44, -2.77
TA SAT (mm)	3.8	0.9	4.3	1.4	3.3	1.8	0.023	c	0.71	0.14, 1.28
MG SAT (mm)	4.0	1.8	5.2	1.9	4.5	3.6	0.226	ns	-0.03	-0.59, 0.53
TA fascicle length (mm)	44.5	12.3	51.2	10.9	65.5	14.9	<0.001	bc	-1.71	-2.34, -1.08
MG fascicle length (mm)	44.9	9.4	50.3	13.6	62.2	11.7	<0.001	bc	-1.58	-2.20, -0.96
TA muscle volume (cm^3^)	61.7	24.1	93.1	39.9	333.0	68.2	<0.001	abc	-8.49	-10.08, -6.91
MG muscle volume (cm^3^)	34.0	12.4	55.6	22.1	156.0	75.2	<0.001	abc	-4.40	-5.34, -3.44
Dorsiflexion torque (Nm)	3.3	3.8	5.2	5.2	33.5	12.9	<0.001	bc	-5.63	-6.76, -4.50
Plantar flexion torque (Nm)	9.1	9.7	16.4	20.7	162.8	63.9	<0.001	abc	-6.55	-7.82, -5.28
Normalized TA muscle thickness	0.08	0.01	0.07	0.01	0.08	0.01	0.008	a	-0.38	-0.94, 0.18
Normalized MG muscle thickness	0.05	0.01	0.06	0.01	0.06	0.01	0.678	ns	0.251	-0.31, 0.81
Normalized TA SAT	0.02	0.01	0.02	0.01	0.01	0.01	<0.001	bc	2.058	1.40, 2.72
Normalized MG SAT	0.02	0.01	0.02	0.01	0.01	0.01	0.001	bc	1.164	0.57, 1.76
Normalized TA fascicle length	0.21	0.07	0.19	0.05	0.17	0.05	<0.001	b	1.00	0.42, 1.58
Normalized MG fascicle length	0.20	0.06	0.19	0.04	0.16	0.03	<0.001	bc	1.18	0.58, 1.77
Normalized dorsiflexion torque	0.18	0.20	0.21	0.23	0.53	0.14	<0.001	bc	-2.32	-3.00, -1.63
Normalized plantar flexion torque	0.54	0.48	0.73	0.85	2.91	0.92	<0.001	bc	-4.13	-5.05, -3.22

a: significant difference between preschool and school children, b: significant difference between preschool children and adults, c: significant difference between school children and adults, ns: no significant difference, BMI: body mass index, TA: tibialis anterior, MG: medial gastrocnemius, SAT: subcutaneous adipose tissue thickness, IQR: interquartile range

#### Muscle morphology

**Fig 1:**
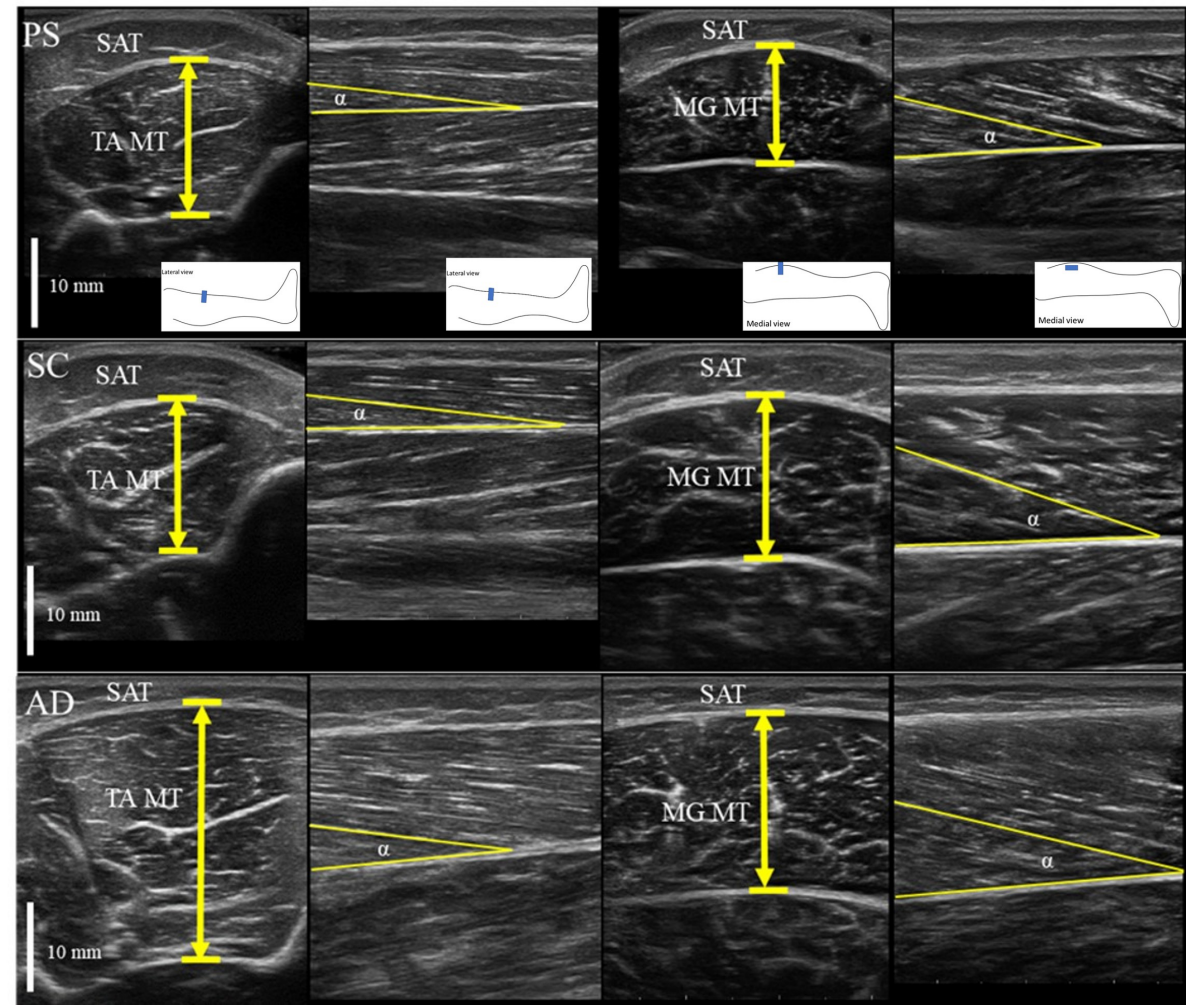
Ultrasonography measurements. (PS: Preschool children, SC: School children, AD: Adult, TA: tibialis anterior, MG: medial gastrocnemius, MT: muscle thickness, SAT: subcutaneous adipose tissue, α: pennation angle).

B-mode ultrasonography (Arietta Prologue, Hitachi, Japan) with a linear array transducer (7.5 MHz scanning frequency) was used to obtain images of the TA and MG muscles for measuring muscle thickness, pennation angle, and fascicle length. Images were obtained at 40% of the proximal shank length for TA and 30% of the proximal shank length for MG, with the ankle joint in a resting neutral position (0 degrees) in supine and prone positions, respectively. The analysis of the ultrasound images was conducted using ImageJ 1.52a software (National Institutes of Health, USA). Muscle thickness was measured using a transverse probe position. Muscle volume was estimated by squaring the muscle thickness and multiplying by the shank length. TA pennation angle was measured as the angle between the central intramuscular septum and the line of the clearest superficial fascicle in the scanned area. The MG pennation angle was calculated as the angle between the fascicle and the deep aponeurosis, with the longitudinal probe position used for measurement. Fascicle length was estimated by dividing the muscle thickness by the sine of the pennation angle, then normalizing it to the shank length. Fig 1 shows an example of ultrasonography and ultrasound probe position for each leg location. Intrarater reliability of muscle thickness and fascicle length measurements was assessed using the intraclass correlation coefficient (ICC_3,1_), with values ranging from 0.860 to 0.991.

#### Strength measurements

**Fig 2:**
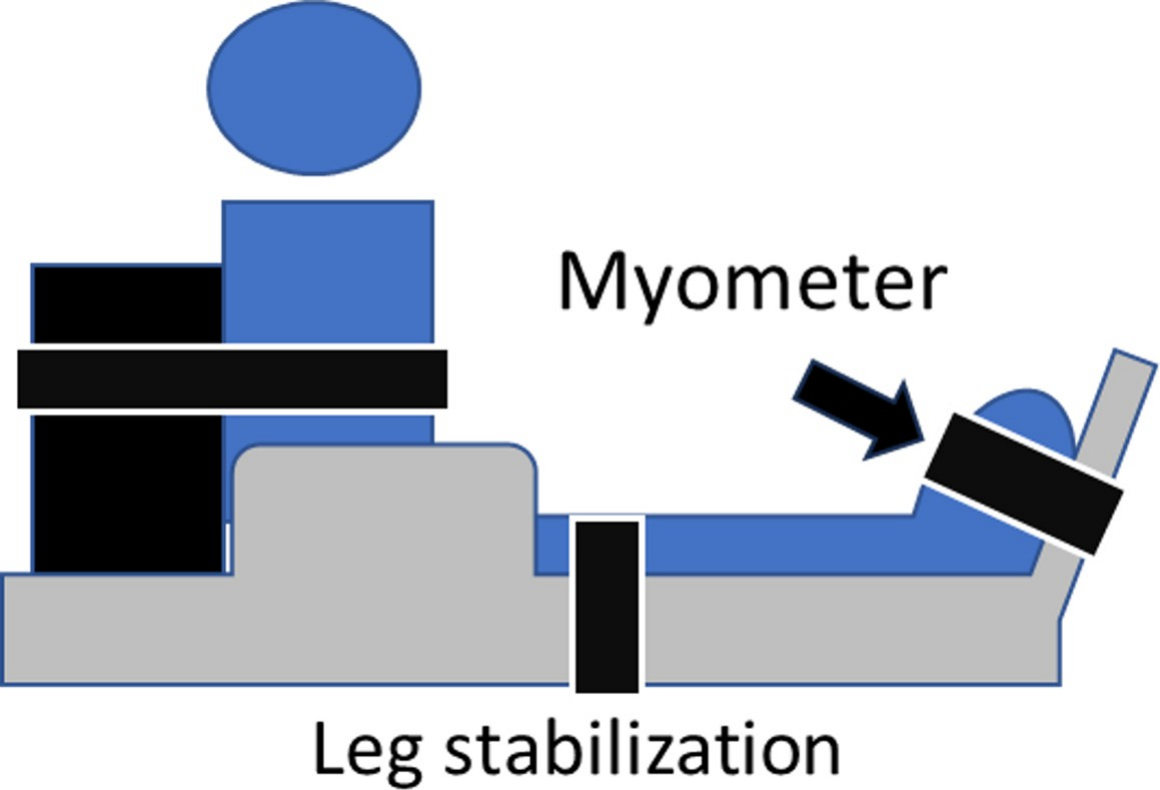
An example of strength measurement (dorsiflexion test).

A myometer (VTK-002, Vine, Japan) and a specially designed myometer (Takei Scientific Instruments, Japan) with leg stabilization were used to measure ankle joint torque in adults and children, respectively. The test was conducted with the knee fully extended and the hip flexed at 90 degrees with non-elastic belt stabilization. The ankle was positioned at the neutral position for the plantar flexion test and at 30 degrees of plantar flexion for the dorsiflexion test. Fig 2 illustrates an example of the position of the dorsiflexion test. Two isometric maximal efforts were performed for 5 seconds with verbal encouragement. Torque values were normalized by individual body mass.

#### Kinematics, ground reaction forces during walking

Walking performance was assessed using a three-dimensional motion analysis system (EvaRT 5.0.4, Motion Analysis Corporation, USA, and KineAnalyzer, Japan), with six cameras (250 Hz) and three force plates (one 9281C, Kistler, Switzerland, and two OR6-5-1, AMTI, USA) with a sampling frequency of 1,000 Hz. Reflective markers (10 mm) were placed on anatomical landmarks (top head, acromion process, lateral epicondyle of humerus, lateral styloid process, distal head of the second metacarpal bone, anterior superior iliac spine, posterior superior iliac spine, greater trochanter, lateral epicondyle of femur, and lateral malleolus) and shoe (distal head of metatarsal bone of the second toe and heel). Lower extremity joint angles in the sagittal plane during the stance phase and GRFs components (including peak force and impulse across three axes) were analyzed. Symmetry indexes for peak force (braking and propulsive peak force), time duration (braking and propulsive duration), and impulse (braking and propulsive impulse) were calculated in the anteroposterior direction as a previous study [22]. GRFs were normalized by body mass and gravitational acceleration, and the normalized GRFs were used to calculate impulse. Spatiotemporal parameters (walking speed, cadence, and step length) were normalized to leg length and gravitational acceleration as described in a previous study [23] using the following equations:

$$\hat{v}=v/\sqrt{gl_{L}}$$ (1)

$$\hat{f}=f/\sqrt{g/l_{L}}$$ (2)

$$\hat{l_{S}}=l_{S}/l_{L}$$ (3)

$\hat{v}$: normalized walking speed

*ν*: walking speed

*g*: the gravitational acceleration

*l*_L_: leg length

$\hat{f}$: normalized cadence

*f*: cadence

$\hat{l_{S}}$: normalized step length

*l*_S_: step length

Lower extremity joint angles were compared among the groups at time points during walking (heel strike, mid-stance, and toe-off), while GRFs were analyzed at the peak of each GRF axis.

#### Muscle activity during walking

During gait analysis, surface electromyography (sEMG) of TA and MG were recorded using a wireless system (Trigno, Delsys Inc, USA). Participant skin was cleaned using 70% isopropyl alcohol solution with soft to medium rubbing) before electrode placement to reduce skin impedance. For adults, if necessary, the area of the skin for electrode placements was shaved before the electrodes were applied. Electrodes were placed on the sites according to SENIAM guidelines (http://seniam.org/) [24, 25] after participants rested for 5 minutes to reduce the skin impedance. Ultrasonography was used to confirm muscle sites to reduce the crosstalk effect. After electrode placement, the correctness of all electrode positions was confirmed through real-time visual inspection of the EMG signals during isolated muscle testing. The sEMG data were selected to analyze from three walking trials with clean foot strikes on the force plate. The sEMG signals were sampled at 1,000 Hz. The raw sEMG data were band-pass filtered with cutoff frequencies at 25 and 450 Hz. The root mean square (RMS) of sEMG was calculated with a window of 50 ms. For each sEMG channel, the highest RMS value (peak RMS) was determined and used for normalization. The co-activation index (CoA) was calculated during the stance phase for TA and MG, with the latter as the agonist [26], using the following equation:

$$CoA=({sEMG}_{an}/{sEMG}_{ag})100$$ (4)

*CoA*: co−activation index

*_s_EMG_an_*: surface electromyography of antagonist

*_s_EMG_ag_*: surface electromyography of agonist

### Statistical analysis

Statistical analysis was performed using SPSS software (Version 24.0, IBM, SPSS Inc., USA). The normality of the data distribution was checked before analysis. Since the data were non-normal distribution, median and interquartile range (IQR) values are presented in Tables 1 and 2. The Kruskal-Wallis test was used to compare differences among the three groups, with Bonferroni correction applied to further analyze significant differences. Spearman’s rank correlation coefficient was used to determine the relationships among variables. The level of significance was set at α = 0.05.

**Table 2:** Biomechanical parameters during shod walking.

	Preschool children		School children			Adults		*p*-value	Sig. (Post hoc)	Cohen’s d	95% C.I.
	Median	IQR	Median	IQR		Median	IQR				
**Spatiotemporal parameters**											
Walking speed (m/s)	1.1	0.2	1.2	0.2	1.5		0.4	0.004	bc	0.971	0.39, 1.55
Stance duration (%)	59.9	3.7	60.9	4.7	60.6		5.9	0.844	ns	-0.143	-0.70, 0.41
Swing duration (%)	40.1	3.7	39.1	4.3	39.4		5.9	0.905	ns	0.064	-0.49, 0.62
Step length (m)	0.5	0.1	0.6	0.1	0.8		0.1	<0.001	abc	-3.17	-3.95, -2.38
Stride width (cm)	8.6	5.1	7.7	4.2	7.3		5.8	0.146	ns	0.366	-0.19, 0.93
Cadence (steps/min)	146.6	18.2	132.3	21.1	108.7		12.2	<0.001	bc	2.041	1.38, 2.70
Normalized walking speed	0.51	0.08	0.49	0.11	0.52		0.16	0.107	ns	-0.128	-0.68, 0.43
Normalized step length	1.08	0.18	1.07	0.17	1.04		0.12	0.077	ns	0.48	-0.08, 1.04
Normalized cadence	0.45	0.08	0.44	0.07	0.51		0.05	<0.001	bc	-1.72	-2.36, -1.09
**Impulse components**											
Medial impulse	0.001	0.002	0.002	0.001	0.002		0.001	0.301	ns	-0.44	-1.01, 0.12
Lateral impulse	-0.015	0.007	-0.015	0.010	-0.013		0.008	0.215	ns	-0.49	-1.03, 0.10
Braking impulse	-0.024	0.015	-0.028	0.008	-0.037		0.014	0.004	bc	1.00	0.42, 1.58
Propulsive impulse	0.024	0.010	0.029	0.007	0.036		0.009	0.038	b	-0.54	-1.10, 0.03
Vertical impulse at early stance	0.214	0.056	0.218	0.029		0.277	0.077	<0.001	bc	-1.32	-1.93, -0.72
Vertical impulse at late stance	0.213	0.074	0.243	0.066		0.263	0.088	0.005	b	-0.92	-1.50, -0.34
Symmetry index of peak force of braking and propulsion	-1.211	0.517	-1.066	0.412		-1.085	0.267	0.485	ns	-0.32	-0.88, 0.24
Symmetry index of duration of braking and propulsion	0.863	0.351	0.857	0.544		1.134	0.227	0.021	bc	-0.82	-1.39, -0.24
Symmetry index of impulse of braking and propulsion	-1.090	0.446	-0.931	0.310		-1.030	0.257	0.786	ns	-0.20	-0.75, 0.36

a: significant difference between preschool and school children, b: significant difference between preschool children and adults, c: significant difference between school children and adults, ns: no significant difference IQR: interquartile range

## Results

### Anthropometric and morphological characteristics of TA and MG, and strength

Table 1 presents anthropometric variables, TA and MG morphology, and dorsi- and plantar flexion strength. Significant differences were observed in muscle thickness, fascicle length, and estimated muscle volume across the three groups, except for fascicle length between the preschool (PS) and school children (SC). Regarding strength, significant differences were found among the three age groups, except for dorsiflexion strength between PS and SC. When normalized by body mass, ankle joint torque was significantly greater in adults (AD) compared to both PS and SC groups.

### Kinematics and kinetics parameters during shod walking

The data on spatiotemporal and impulse parameters are presented in Table 2. Walking speed, step length, and cadence showed significant differences between the children and AD. After normalizing the variables, only cadence was significantly different between the two children groups and AD. Impulses in the anteroposterior and vertical directions were greater in AD than in children, particularly between AD and PS. The symmetry of the duration of the braking and propulsion phases showed significant differences between children and AD, while other symmetry indices were comparable across all groups.

**Fig 3:**
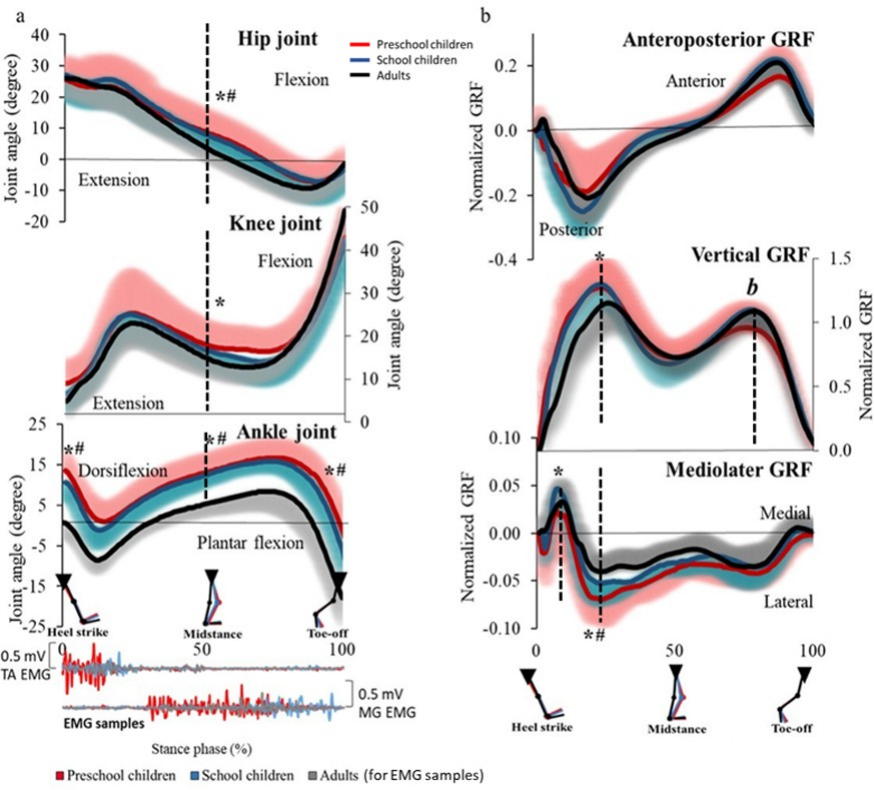
Biomechanical patterns during the stance phase. a) lower extremity joint angle patterns and lower leg EMG samples b) ground reaction force patterns, The solid line represents the mean, and the shaded area represents the standard deviation, TA: tibialis anterior, MG: medial gastrocnemius, GRF: ground reaction force, *: significant difference between preschool and adults, #: significant difference between school children and adults, b: significant difference between preschool and school children (For kinematics, comparisons were performed at heel strike, mid-stance, and toe-off. While GRFs were analyzed regarding the peak of each axis).

Fig 3 illustrates the changes in the hip, knee, and ankle joint angles, along with the normalized GRFs components. At mid-stance, the hip joint angle was significantly larger in the children’s groups compared to AD, while the knee joint angle differed only between PS and AD. Ankle joint angles were significantly different in both PS and SC compared to AD at heel strike, mid-stance, and toe-off. Regarding kinetics, the vertical peak force at early stance was generally greater in children than in AD, while the vertical peak force at late stance was lower in PS compared to SC and AD. The lateral peak force at early stance was significantly higher in PS and SC than in AD. Lower extremity joint angle and normalized ground reaction force parameters during shod walking are shown in Tables S1.1 and S1.2, respectively (S1 File).

### TA and MG muscle activity during the stance phase

**Fig 4:**
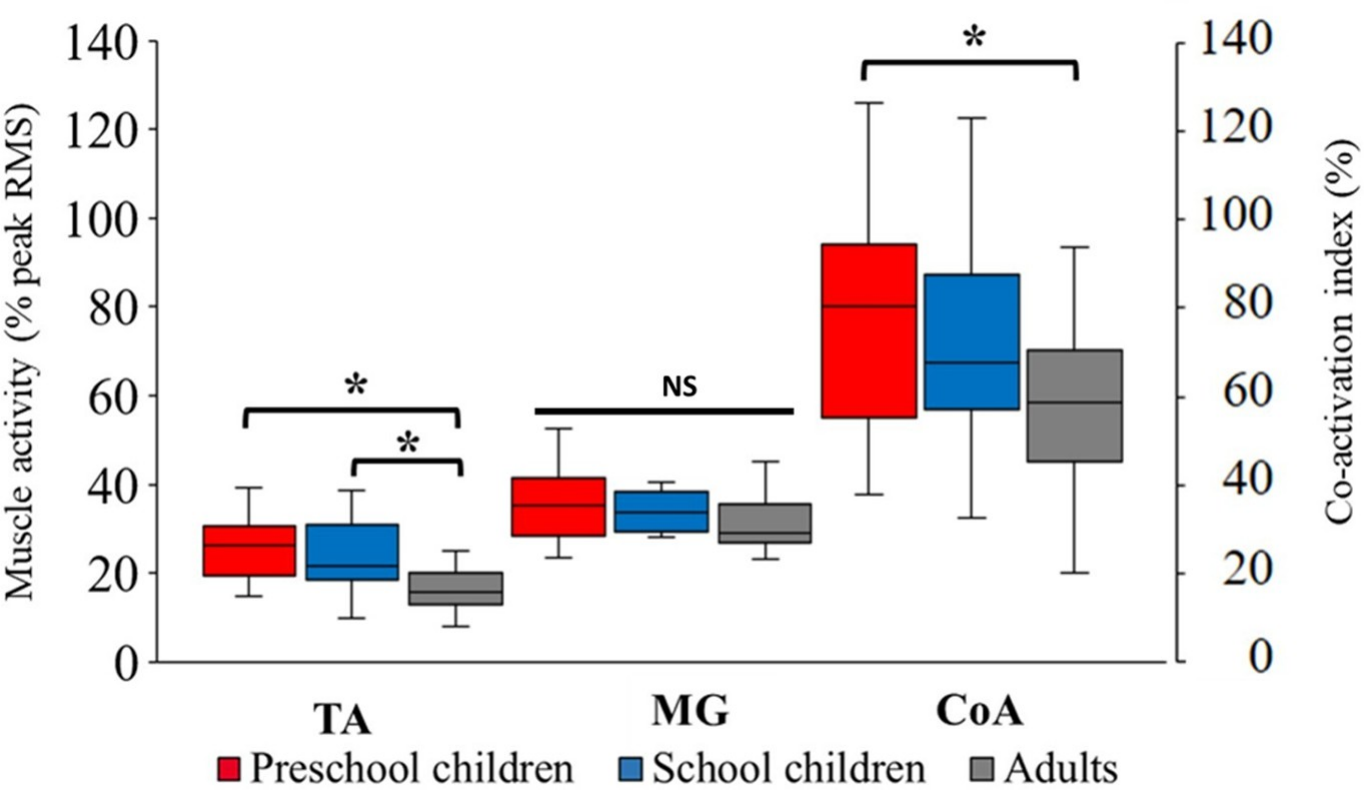
Muscle activity patterns. TA: tibialis anterior, MG: medial gastrocnemius, CoA: Co-activation index, *: significant difference, NS: no significant difference.

The TA and MG muscle activities during the stance phase are shown in Fig 4. Relative TA activity was significantly greater in PS and SC compared to AD, while no significant differences were observed for MG among the groups. CoA during the stance phase (TA/MG) was significantly greater in PS than in AD. Muscle activity and co-activation index parameters during shod walking are shown in Table S1.3 (S1 File).

### Correlations of walking performance with age and muscle morphological characteristics

**Fig 5:**
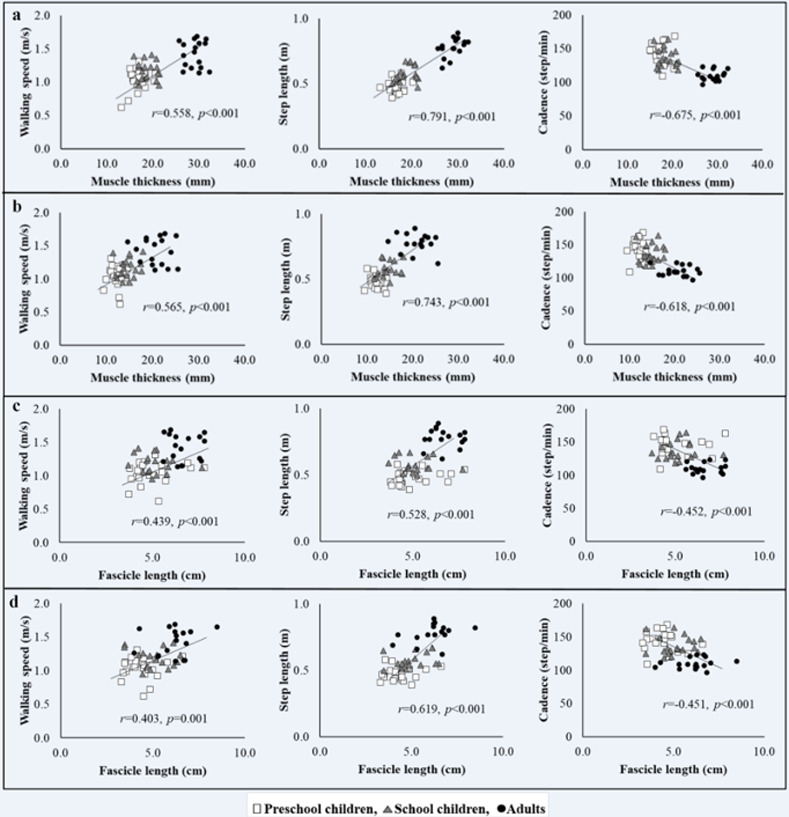
a) TA muscle thickness and walking performance, b) MG muscle thickness and walking performance, c) TA fascicle length and walking performance, d) MG fascicle length and walking performance.

The relationship between age and walking speed was significant only in PS (*r* = 0.456, *p* = 0.022). Mild to moderate associations were observed between age and step length in PS (*r* = 0.565, *p* = 0.004) and SC (*r* = 0.493, *p* = 0.038), respectively. Additionally, walking speed was associated with cadence in both PS (*r* = 0.480, *p* = 0.038) and SC (*r* = 0.573, *p* = 0.010). Lower leg muscle size and fascicle length were significantly correlated with walking performance when the groups were pooled (Fig 5A–5D).

## Discussion

The purpose of this study was to investigate the anatomical, biomechanical, and muscle activation patterns and their relevance to shod walking performance in PS, SC, and AD. Our results reveal age-related trends in muscle growth and development, which significantly effected walking performance, particularly in PS. Walking performance at a preferred speed was also associated with lower leg muscle thickness and fascicle length. Furthermore, our findings indicated greater muscle co-activation around the ankle joint in children compared to adults and the specific walking patterns of PS and SC. These findings partly support the previous knowledge of the ankle stabilization pattern in young children [4, 6]. Muscle co-activation around ankle joints increased in children to stabilize foot weight acceptance [27], which may influence the impact loading on the tibia during stance. A previous study suggested that tibial shock attenuation is a part of the mechanism for maintaining body stabilization [28]. This may indicate a higher demand for motor control strategies in children. However, greater muscle co-activation during stance might also suggest a less robust gait strategy [29]. The previous study proposed that the increased number of muscle synergies in children may be linked to motor learning [30].

Our findings indicated muscle growth (size and architecture) for both the anterior and posterior parts of the lower leg. Significant group differences were evident in TA and MG thickness and volume in absolute terms, aligning with previous studies showing notable lower extremity muscle size differences between children and AD [11, 12, 20]. In this study, estimated muscle volume was calculated using muscle thickness and shank length that may not reflect the muscle shape in three-dimension. Future research should consider more precise measurement techniques, although prior work has validated that muscle thickness is correlated with muscle cross-sectional area and volume [31]. Fascicle lengths were shorter in children compared to AD, which is reasonable given that fascicle length and pennation angle correlate with muscle thickness [18, 32]. In relative terms, such inter-group differences show complicated tendencies, sometimes suggesting that muscle morphological growth is dependent on the body size regardless of age, while in other cases the tendency is on the contrary, e.g., PS had relatively thicker muscle (TA) and longer fascicles (TA and MG) than those in SC and AD. Furthermore, these relative results are not parallel to those of joint torque, suggesting that strength in children in particular is not a simple function of their muscle size. Regardless of muscle size, there were clear strength differences between children and AD.

Our study revealed that absolute walking performance measures were generally but not always greater with advancing age. Among parameters with clear group differences, step length, a key determinant of walking speed, was positively related to lower leg muscle size. In contrast, the cadence was not related to muscle morphology, and children (PS in particular) showed higher cadence. The rhythm of walking may be influenced by motor skills and developments rather than body dimensional growth [33, 34]. Regarding the biomechanical aspects of walking, for GRFs, PS showed a tendency for lower braking and propulsive forces compared with SC and AD, but no significant differences were found among the three groups. The impact peak force was greater in PS and SC compared with AD, while the vertical peak force in the pre-swing was lower in PS than in SC and AD. Additionally, there were significant group differences in vertical and anteroposterior impulses, as well as in the symmetry index of braking and propulsion duration. Previous research has shown that adults exhibit greater GRFs for braking, propulsion, and vertical force at pre-swing compared with young children during barefoot walking [6], findings that are partially but not entirely aligned with our results. Although participants were familiarized with the shoe before the walking session, the ankle range of motion at heel strike was greater in children than in AD. These impact characteristics may result from higher heel strike patterns [35], especially in habitually barefoot children. The reasons for the lower GRF at pre-swing in children may be due to their immature foot arch and plantar flexor muscles which render rolling movement of the foot during stance [4, 36, 37]. For the mediolateral GRF, the children showed lateral force patterns at early stance and loading response to mid-stance, especially in PS. A trend of stride width decreasing with age was also found in the study. These findings are in agreement with previous reports that mediolateral force and stride width decreased with age [10, 38]. We could point out that the biomechanical aspects of shod walking are close to the adult-like pattern from SC. In the first half of the stance phase, PS demonstrated specific GRFs characteristics with the greater impact force and lateral force pattern, and vice versa the lower peak forces of propulsion and vertical direction in the second half of the stance phase.

Concerning motor control, our findings indicate that walking at a self-selected speed requires greater effort in early childhood than in AD, particularly for the TA. Unlike adults, children may not yet have fully developed the neuromuscular strategies necessary to achieve efficient gait patterns. Given the difficulty younger children experience with motor control during maximum voluntary contraction (MVC) tests, we normalized sEMG signals to the peak RMS during each trial. In children, TA activity during the stance phase may approach this peak RMS, unlike in adults, who can regulate muscle activity with greater efficiency. A previous study showed that torque-producing capacity was highly variable in children, who can exert much smaller maximal torque per muscle volume compared to AD [20], which may be the reason for the greater effort during walking. Interestingly, a co-activation of TA and MG during the stance phase was greater in children than in AD, especially in PS. The increased muscle co-activation can accompany the reduction of agonist activity regardless of antagonist activation [26]. However, in this study, the higher co-activation in children was due to the higher activity of TA (antagonist). The CoA and its standard deviation in children were higher than those observed in adults. Additionally, the TA activity (%Peak RMS) in some children appeared to exceed the MG activity (%Peak RMS) compared to adults. This discrepancy may account for the standard deviation exceeding 100% (Fig 4). These findings potentially reflect the children’s voluntary effort to maintain their ankle joint angle, characterized by increased TA activity during the early stance phase. Although hip and knee joint motion patterns demonstrated an adult-like pattern in PS, it was not the case for the ankle. In PS and SC, the ankle was more into dorsiflexion during the whole stance phase, unlike in AD. AD showed a greater range of plantar flexion for propulsion, unlike small children who were co-activating antagonist/agonist muscles to stabilize the ankle during the stance phase. Additionally, other potential factors contributing to child-adult differences in muscle activation may include muscle fiber type composition, intra-muscular synchronization, and motor unit activation [39].

### Limitations

This study has several limitations. First, we did not examine sex differences in muscle morphology and walking performance, based on previous findings that muscle morphology is generally not sex-dependent in children aged 5–12 years [40] and the sex influenced the gait patterns only in the period of adolescence as a result of the maturity of musculoskeletal and neurological systems [41, 42]. Second, substantial variability was observed in kinematics and ground reaction forces (GRFs), particularly among children. This variability could reflect an increased energy cost of walking due to greater co-activation of ankle muscles. Further analysis of individual biomechanical patterns by age range may help clarify these age-specific differences. Third, the younger participants faced challenges in performing the Maximal Voluntary Contraction (MVC) test, likely due to limitations in motor control development of the ankle function. Consequently, sEMG signals were normalized by peak RMS values during trials rather than by MVC, which may impact the interpretation of muscle activation. Fourth, walking at different speeds could affect biomechanical patterns, especially in young children. Further studies are warranted to examine these effects under controlled speed conditions. Fifth, two distinct force plate systems were used in our study, which may have influenced the results despite careful calibration of all gait analysis equipment. Sixth, muscle volume in this study was estimated using a simplified method that involved squaring the muscle thickness and multiplying it by the shank length. Future studies should explore more precise measurement techniques. Lastly, the small sample size in each age group and the use of self-selected walking speeds may have contributed to individual differences and variability within groups. Future studies would benefit from greater control over experimental equipment and environmental conditions to reduce variability.

## Conclusions

Walking speed is a function of lower leg muscle morphology (thickness and fascicle length) which is reflected in step length and cadence. The relative maximal strength is a function of age; however, it does not affect walking performance. In early childhood, particularly among PS, walking requires greater, as indicated by a lateral stepping pattern and notable co-activation of lower leg muscles, even at a self-selected speed. Future studies should examine this co-activation strategy across various walking speeds and with larger sample sizes to confirm these developmental gait characteristics.

## Supporting information

S1 File (XLSX)
